# The coral *Platygyra verweyi* exhibits local adaptation to long-term thermal stress through host-specific physiological and enzymatic response

**DOI:** 10.1038/s41598-019-49594-z

**Published:** 2019-09-17

**Authors:** Jih-Terng Wang, Yi-Ting Wang, Shashank Keshavmurthy, Pei-Jei Meng, Chaolun Allen Chen

**Affiliations:** 1 Department of Biotechnology, Tajen University, Pingtung, 907 Taiwan; 2 Biodiversity Research Center, Academia Sinica, Taipei, 115 Taiwan; 3 National Museum of Marine Biology and Aquarium, Pingtung, 944 Taiwan; 4 Institute of Marine Biodiversity and Evolution, National Dong Hwa University, Pingtung, 944 Taiwan; 5 Institute of Oceanography, National Taiwan University, Taipei, 108 Taiwan; 6 Department of Life Science, Tunghai University, Taichung, 404 Taiwan

**Keywords:** Enzyme mechanisms, Marine biology

## Abstract

Climate change threatens coral survival by causing coral bleaching, which occurs when the coral’s symbiotic relationship with algal symbionts (Symbiodiniaceae) breaks down. Studies on thermal adaptation focus on symbionts because they are accessible both *in vitro* and *in hospite*. However, there is little known about the physiological and biochemical response of adult corals (without Symbiodiniaceae) to thermal stress. Here we show acclimatization and/or adaptation potential of menthol-bleached aposymbiotic coral *Platygyra verweyi* in terms of respiration breakdown temperature (RBT) and malate dehydrogenase (MDH) enzyme activity in samples collected from two reef sites with contrasting temperature regimes: a site near a nuclear power plant outlet (NPP-OL, with long-term temperature perturbation) and Wanlitong (WLT) in southern Taiwan. Aposymbiotic *P. verweyi* from the NPP-OL site had a 3.1 °C higher threshold RBT than those from WLT. In addition, MDH activity in *P. verweyi* from NPP-OL showed higher thermal resistance than those from WLT by higher optimum temperatures and the activation energy required for inactivating the enzyme by heat. The MDH from NPP-OL also had two times higher residual activity than that from WLT after incubation at 50 °C for 1 h. The results of RBT and thermal properties of MDH in *P. verweyi* demonstrate potential physiological and enzymatic response to a long-term and regular thermal stress, independent of their Symbiodiniaceae partner.

## Introduction

Reef-building corals are generally distributed in tropical oceans with stable seawater temperatures and low nutrients and make up one of earth’s complicated but highly productive ecosystems^1^. The success of scleractinian corals in oligotrophic seawater is primarily attributed to their association with dinoflagellate algae^1^, previously assigned to the genus *Symbiodinium* and recently changed to family Symbiodiniaceae^2^. This association is highly sensitive to climate change-induced rising seawater temperatures^3^. A mere 1~2 °C increase in summer average seawater temperatures coupled with moderate to high irradiance disrupts this symbiotic relationship by expelling the symbiotic algae from the coral host, resulting in so-called coral bleaching^4,5^. Increased incidences of repeated above-threshold seawater temperature has led to more frequent coral bleaching events covering wider areas—for example, the Great Barrier Reef^6^ in recent years—further highlighting the threat of thermal impact on coral survival and thus evoking intensive attention to the coral’s ability and mechanisms for adapting to a warming environment.

The mechanisms that underpin coral adaptation to rising temperatures are more complicated than in the other aquatic organisms because of the holobiont nature of corals, wherein in addition to their symbiosis with Symbiodiniaceae, they are also associated with a multitude of other microbes^7^. Current understanding of how corals respond to thermal stress has shown that at least some species and/or populations have the capacity to acclimatize and/or adapt to warmer conditions by shifting to acquire algal species with a higher thermal tolerability, or by adjusting the physiological performance or genetic structures of coral hosts (for details see the review^8^ and references therein). Surveys of different Symbiodiniaceae species have also suggested that some of them (e.g., *Durusdinium trenchii*) are more capable of preventing or repairing photosynthetic damage induced by thermal stress^9–12^. Many ecological studies have also confirmed that the resistance of corals to thermal bleaching is highly correlated with the abundance of thermal-tolerant algal species and a greater prevalence of *D. trenchii* in several bleaching resistant or warm-water symbioses^13–22^ and in some cases prevalence of certain species of *Cladocopium* (for example, in corals present in Persian/Arabian Gulf^23^).

Despite the breadth of knowledge on Symbiodiniaceae algae, what we know of how coral hosts respond to thermal stress is derived merely from the studies on coral holobionts (coral + symbionts including bacteria) and non-symbiotic larvae^24–27^, but not on aposymbiotic (in this paper this means without Symbiodiniaceae) adult coral colonies due to the lack of an algal-exclusive culture technique. However, several lines of evidence from the studies on coral holobionts have also suggested that coral hosts might respond to heat stress by regulating photo-protective and antioxidant systems^4,28^, increased heterotrophy^29^, and symbiont cell densities^30^, as well as associated bacterial community (see^31^). Coral hosts are also capable in upregulating the expression of stress-related genes (e.g., heat shock proteins and ROS scavengers) under heat stress^24,25,32,33^. Given that corals are long-lived organisms, the role of individual acclimatization rather than genetic adaptation was widely expected to play a leading role in their response to global warming^24^. However, inter-latitudinal crosses of coral parents from warmer and cooler locations clearly demonstrated thermal tolerance of coral is heritable and evolvable^25,34,35^. In addition, a high correlation between thermal tolerance and genetic changes was observed at a number of loci within the same population of *Acropora hyacinthus* that inhabited different pools with high and moderate temperature variation and no dispersal barriers in between^36^. Though genomic evidence strongly supports temperature adaptation in corals, no information has been presented to directly link protein adaptation in coral hosts to thermal stress, which has been widely described in the other aquatic organisms^37–42^.

Temperature is suggested to be a major driving force in evolution^43^. Accordingly, organisms are expected to adjust their physiological or enzymatic performance to acclimatize and/or adapt to the effects of elevated temperature^43^. It has been suggested that physiological performance is a powerful approach to examine the evolution of thermal tolerance^44^. Many studies have demonstrated that respiration performance is a feasible physiological indicator of thermal acclimatization to climate changes in marine invertebrates^45^, fishes^46^, plants^47^ and soil microbes^48^. Moreover, at the molecular level, enzyme activity is closely linked to metabolic rate and food availability in fishes and marine invertebrates^45–49^. Temperature adaptation of proteins, especially the enzymes critical to energy metabolism, have been documented in many aquatic ectothermic taxa inhabiting a wide range of thermal habitats^37–42^. Studies have shown that only one or two amino acid substitutions is enough to increase thermal stability in malate dehydrogenase (MDH)^42^, isocitrate dehydrogenase (IDH)^50^ or lactate dehydrogenase (LDH)^51^. A recent study^52^ further demonstrated that protein adaptation to temperature could be quantified by applying a molecular dynamics simulation to analyze the degree of a protein’s structural flexibility.

Herein we demonstrate, in an aposymbiotic coral host (artificially bleached using menthol) its potential physiological and enzymatic response to long-term thermal stress. We compared respiratory physiology and enzyme characteristics of MDH in a menthol-treated aposymbiotic brain coral, *Platygyra verweyi*^53^, between a reef located adjacent to a nuclear power plant outlet (NPP-OL, 2~3 °C elevated seawater temperature) and a nearby reef at Wanlitong (WLT) in the Kenting National Park, southern Taiwan. NPP-OL is a natural mesocosm with seawater temperature similar to the scenario forecasted for ocean temperatures in 2050^25^.

Previous studies have shown that the coral assemblage composition in the shallow reef (<3 m in depth) near NPP-OL not only became dominated by stress-tolerant species, but also to had concurrent associations with the tolerant Symbiodiniaceae belonging to *D. trenchii*.^20^. *Platygyra verweyi* is one of the stress-tolerant species associated primarily with *D. trenchii (*formally type D1a) nearby NPP-OL, and with *Cladocopium* C3 at WLT^54^. *P. verweyi* shows location-based specificity with the type of Symbiodiniaceae genera it associates with^19^. Studies also have shown that there is no genetic differentiation^19,54^ between the hosts within and between NPP-OL and WLT in the Kenting National Park, which implies that coral host-symbiont combinations are responsible for coral’s long-term acclimatization and/or adaptation^19,51,54^. Location-based specificity in association with different symbionts also makes hosts associated with *Cladocopium* C3 susceptible to bleaching and mortality during prolonged temperature stress^54^. So, there might be other mechanisms, such as host-specific responses through physiological or enzymatic performance, involved in the local acclimatization and/or adaptation of *P. verweyi-*symbiont combinations.

## Materials and Methods

### Study sites, sample collection, and aposymbiotic corals by menthol bleaching

Brain coral, *P. verweyi*, was sampled at a depth of 2–3 meters from 2 locations; one next to the 3rd nuclear power plant outlet (NPP-OL, 120°44′13″E, 21°56′4″N) and the other at Wanlitong (WLT, 120°41′43″E, 21°59′5″N) in the Kenting National Park, Taiwan. The reef at NPP-OL has experienced 2–3 °C higher seawater temperature than the ambient environment every summer since 1984 due to the operation of the 3rd NPP. In addition, due to the tidally-induced upwelling in Nanwan^55^, the maximum daily seawater temperature fluctuation at NPP-OL can be more than 8 °C in the summer^54^. In contrast, WLT, located on the west coast of the Kenting National Park, has an average summer (June to August) seawater temperature similar to NPP-OL, while the intervals of extreme temperature events (≥30 °C) and daily seawater temperature fluctuations are shorter and less extreme than NPP-OL^56^. Collected corals were acclimated in an aquarium maintained at the conditions as described previously^53^ for at least 4 days before use.

The menthol-bleaching method^53^ was used to create aposymbiotic corals. Briefly, small fragments of corals were incubated in 300 ml menthol-supplemented artificial sea water (ASW, Instant Ocean, Aquarium Systems, Sarrebourg Cedex, France). The menthol/ASW medium was prepared by diluting a 20% (w/v) menthol stock (in ethanol) with ASW. Incubation in menthol was ended (at 3 weeks) when the coral was completely bleached (Fig. S1). The bleached coral host samples were used to determine the respiration breaking temperature within a week.

In order to collect enough enzyme for final kinetic analysis, more than 10 runs of purification process were carried out. At each run of purification, nubbins (approximate size = 7 × 7 cm) from 7–8 different colonies were used. Since enzyme kinetic analysis requires large amount of purified enzyme, which is almost not possible for a coral colony to provide, and each purification process is time consuming process, the experiment did not have any biological replicates as well as controls.

### Respiration breaking temperature (RBT) of coral host

**Figure 1:**
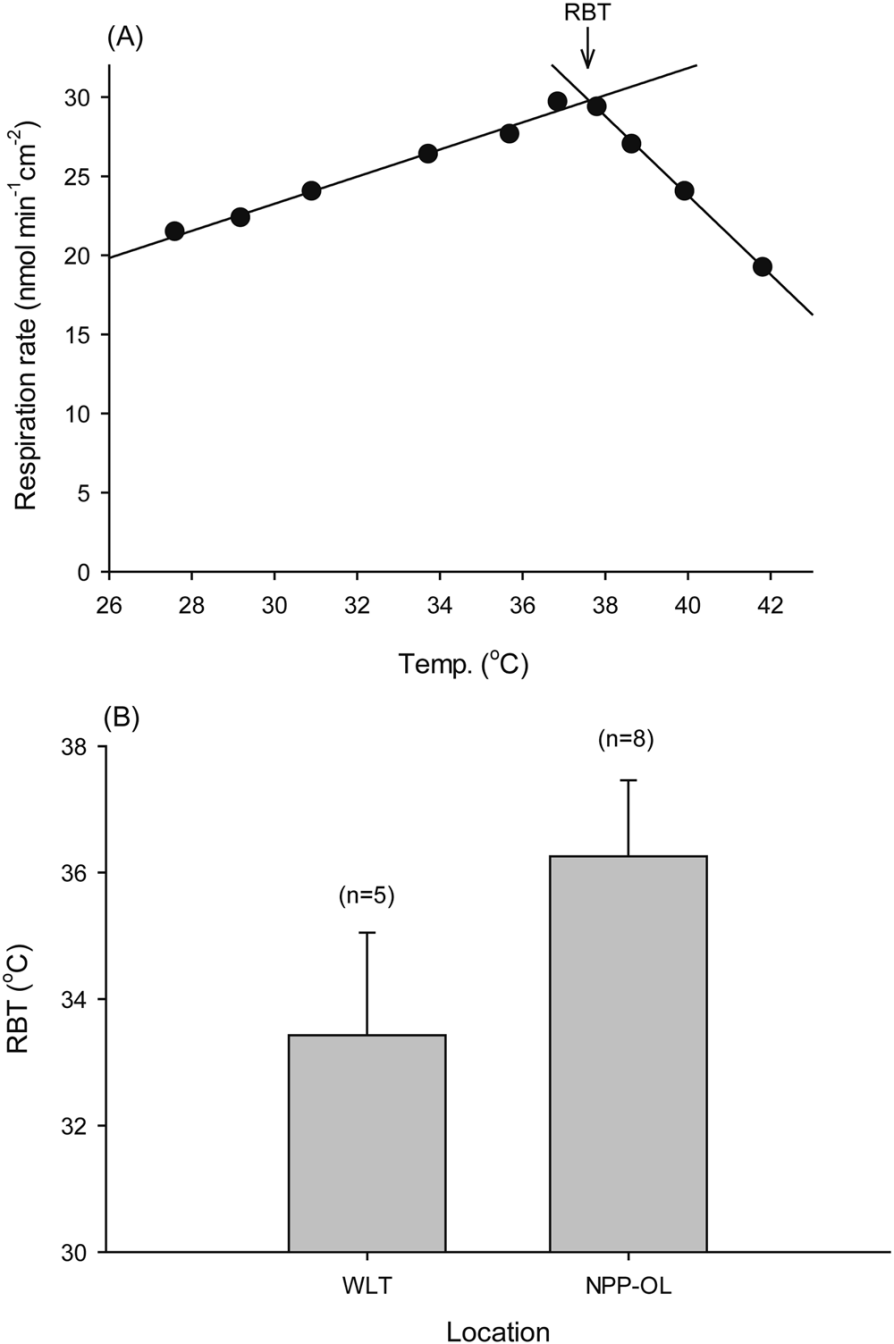
Respiration response of aposymbiotic *Platygyra verweyi* to temperature elevation. (**A**) An example plot of the changes in respiration rate at different incubation temperatures, in which the respiration breaking temperature (RBT) was determined by the intercept calculated from the linear regression equations of increasing and decreasing respiration rate on temperatures. (**B**) RBT of the corals collected from a reef near the nuclear power plant outlet (NPP-OL) and an ambient location, Wanlitong (WLT), away from the thermal effluence. The RBT values are shown as mean ± S.D. Numbers of replicated colonies were listed on top of each bar.

Aposymbiotic coral nubbins were subjected to heat treatment by incrementally increasing its temperature. Coral nubbins were incubated in a respiratory chamber immersed in a water bath at room temperature for 10 min. Heat treatment was initiated by transferring coral nubbins from room temperature directly into another respiration chamber with preheated (about +2 °C) and aerated artificial seawater (ASW) medium. At each temperature treatment, coral nubbins were incubated for 15 min (5 min for temperature equilibrium and 10 min for oxygen consumption measurement) before being transferred to the next higher temperature treatment. The heating process was ended at 42–43 °C. ASW temperature was measured to 0.01 °C precision with a digital thermometer (TM-907A, Lutron, Taiwan) connected to a thermocouple (TP-100, Lutron, Taiwan). While measuring oxygen consumption, ASW medium in the respiration chamber was mixed with an underwater magnetic stirrer. Changes in oxygen content were measured with an optical dissolved oxygen instrument (YSI ProODO, USA) to avoid electrode polarization, and the signal was channeled to a computer for data collection. RBT was determined by calculating the intercept of two linear regression lines developed respectively from the increase and decrease in respiration rate when the temperature was elevated (Fig. 1A).

### Purification of malate dehydrogenase (MDH)

To answer if the differences in thermal sensitivity of respiration physiology between the coral samples from NPP-OL and WLT was linked to the modification of enzymes, MDH, a key enzyme in aerobic energy metabolism in *P*. *verweyi*, host tissue extract was analyzed with isozyme composition and Kinetic performance to temperature. MDH was isolated by blasting symbiotic coral with ice-cold ASW and homogenizing the tissue slurry with a hand-held glass tissue grinder. The resulting homogenate was centrifuged at 4 °C (x15,000 rpm) for 15 min to separate symbiotic algae and tissue debris, then the fraction was collected in 30–80% saturation of ammonium sulfate, re-suspending in 3.2 M (NH_4_)_2_SO_4_ containing 80 mM phosphate buffer (pH 7.5) and 0.1 M EDTA, and storing at 4 °C before use.

The isoforms of MDH were examined with a diethylaminoethanol (DEAE) Sepharose^TM^ (Fast Flow, GE healthcare) column (2.6 × 30 cm), and the major MDH fraction was further purified following the modified methods described in^57,58^. Practically, MDH was purified with a serial column chromatography, including DEAE, CL-6B column, hydroxyapatite, and Sephacryl S-200 column. The detail chromatography condition is described in the supplement. MDH activity in the eluate was measured at 25 °C by adding 50 µl enzyme solution into a substrate solution mixed with 50 µl 3 mM NADH, 50 µl 1 mM oxaloacetate (OAA) and 900 µl 100 mM phosphate buffer pH 7.0. The enzyme activity was determined by the decrease in 340 nm absorbance recorded by time scanning mode in a spectrophotometer (Hitachi U1900) and standardized with protein concentration determined with Bradford Assay. The changes in 280 nm absorbance and salt concentration of the eluate gradient were determined with a spectrophotometer (Hitachi U1900) and a refractometer, respectively.

SDS-PAGE in 12.5% gel visualized with coomassie blue staining was used to determine the purity and molecular weight of MDH in the Sephacryl S-200 eluate. The molecular weight of native MDH was determined by Sephacryl S-200 gel filtration after comparing with a set of purified protein markers included ribonuclease A (13.7 kDa), chymotrypsinogen A (25 kDa), ovalbumin (43 kDa,) and albumin (66.5 kDa). Protein concentration was determined by a Bradford Assay (Bio-Rad Protein Assay, Bio-Rad) according to the manufacturer’s protocol.

### Effects of temperature on enzyme kinetic parameters and stability

The enzyme reaction was determined as described in the purification section, except that OAA concentration was varied from 14 to 29 μM with a fixed NADH concentration (3 mM) to determine OAA’s substrate concentration (*K*_m_) and saturating substrate concentration (*V*_max_), and NADH varied from 38 to 95 µM with a fixed OAA concentration (1 mM) to determine NADH’s *K*_m_ and *V*_max_. All reaction rates were obtained from the mean of triplicate reactions. The reaction temperature in the cuvette was maintained using a water jacket cuvette holder connected to a thermostatic bath (YIH DER, BL-720). Prior to each thermodynamic analysis, a digital thermometer was used to confirm temperature stability in the cuvette three times when mixing 100 µl substrate, 50 µl enzyme and 900 µl buffer (maintained in a thermostatic bath at the reaction temperature).

Obtained *V*_max_ values were further used to calculate the activation energy required for MDH catalysis (Ea_cat_) and that for inactivating MDH activity (Ea_inact_) by temperature with the Arrhenius equation. Ea_cat_ and Ea_inact_ values were usually used to evaluate the thermostability of enzymes in different organisms. The Ea_cat_ was determined by plotting natural-logarithmically (ln) transformed *V*_max_ on T^−1^ in °K, in which the *V*_max_ data were obtained from pre-optimum temperature. The Ea_inact_ was calculated by plotting the ln-transformed inactivation rate constants *k* on T^−1^ in °K. The *k* values were determined by subtracting each *V*_max_ obtained at the post-optimum temperature from the *V*_max_ at optimum temperature and converted to per second. The fitness of the kinetic data to the Arrhenius equation, [ln (*V*_max_ or *k*) = ln(A) − (Ea/R)(1/T)], was examined by a linear regression of ln(*V*_max_ or *k*) against T^−1^ (R = 8.314 J mol^−1^ °K^−1^).

The thermal stability of MDH isolated from NPP-OL and WLT coral samples were compared by incubating the diluted enzyme solution (in 100 mM phosphate buffer pH 7.0) containing activity of about 20 nmol min^−1^ at 50 °C for 60 min. Residual activities of MDH in percentages were determined with the ratio of MDH activity at each time interval compared to the original. Triplicate measurements of enzyme activity at every time interval were made under the condition described in the purification section.

### Statistical analysis

Respiration performances of *P. verweyi* coral hosts between NPP-OL and WLT were compared with a Student’s *t*-test. Differences in the slopes of linear regression of ln(*V*_max_), ln(*K*_m_) and ln(*k*) on temperature in °K were analyzed by Minitab statistical software.

## Results

### Thermal experience shapes the response of coral host respiration to temperature

The respiration rate of aposymbiotic *P*. *verweyi* initially rose linearly when incubation temperature was increased, but started to decrease at higher temperature when reaching the RBT (Fig. 1A). All datasets showed high regression coefficient r^2^ values, 0.970 ± 0.024 (mean ± SD, N = 10) and 0.982 ± 0.018 (N = 16) for NPP-OL and WLT respectively. The mean RBT of *P. verweyi* from NPP-OL samples was 36.7 ± 1.4 °C (mean ± SD, N = 5), which was significantly higher than the 33.6 ± 2.0 °C (N = 8) from WLT (*t*_11_ = 2.9153, P < 0.05, df = 11) (Fig. 1B).

### MDH chromatography pattern and purity

**Figure 2:**
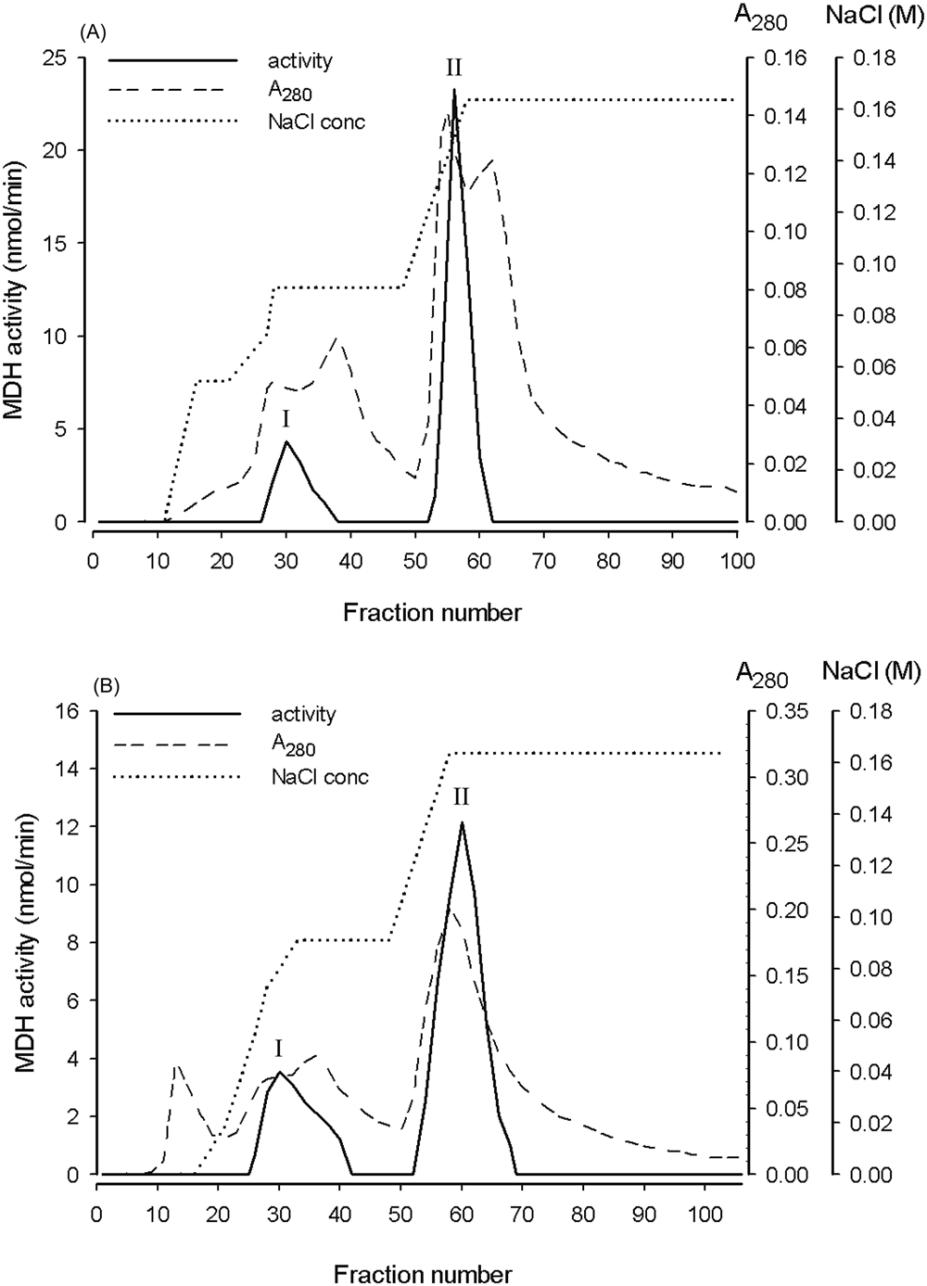
DEAE chromatography of malate dehydrogenase in the coral tissue extract of *Platygyra verweyi* from (**A**) NPP-OL and (**B**) WLT. Peak II was collected for further purification.

Both NPP-OL and WLT samples displayed a consistent profile that contained only two MDH fractions (Fig. 2). The two MDH fractions could not be further separated using a linear gradient of different NaCl concentrations (data not shown). Thus, only the major MDH isozyme (peak II) which contained >75% of total activity was collected for further purification. MDH in the DEAE eluate from both NPP-OL and WLT samples displayed only one peak and almost identical elution conditions in the following Sepharose CL-6B, hydroxyapatite and Sephacryl S-200 column chromatographies (Figs S2–S4). In the Sephacryl S-200 gel chromatography, the MDH activities of both NPP-OL and WLT samples were eluted synchronously with A_280_ absorbance, indicating that the enzymes had been highly purified at both sites. MDH purity in the Sephacryl S-200 eluate was estimated to be >90% by SDS-PAGE and coomassie blue stain, as shown in Fig. S4, and only one clear band was visualized at about 39 kDa for the NPP-OL sample and 38 kDa for the WLT one. With calibration to standard proteins by Sephacryl S-200 gel filtration (Fig. S5), the molecular weight of the native MDH form was about 67 kDa and 64 kDa for the NPP-OL and WLT samples, respectively, indicating that the major MDH of *P. verweyi* was a typical dimer. Finally, the overall purification process described above increased the specific MDH activities from about 2 µmol min^−1^ mg^−1^ in the tissue extract to 77 (from NPP-OL) and 111 µmol min^−1^ mg^−1^ (from WLT) in the Sephacryl S-200 eluates; these purified enzyme solutions were used for further kinetic analysis at the different temperatures.

### Enzyme kinetics and thermal susceptibility

**Figure 3:**
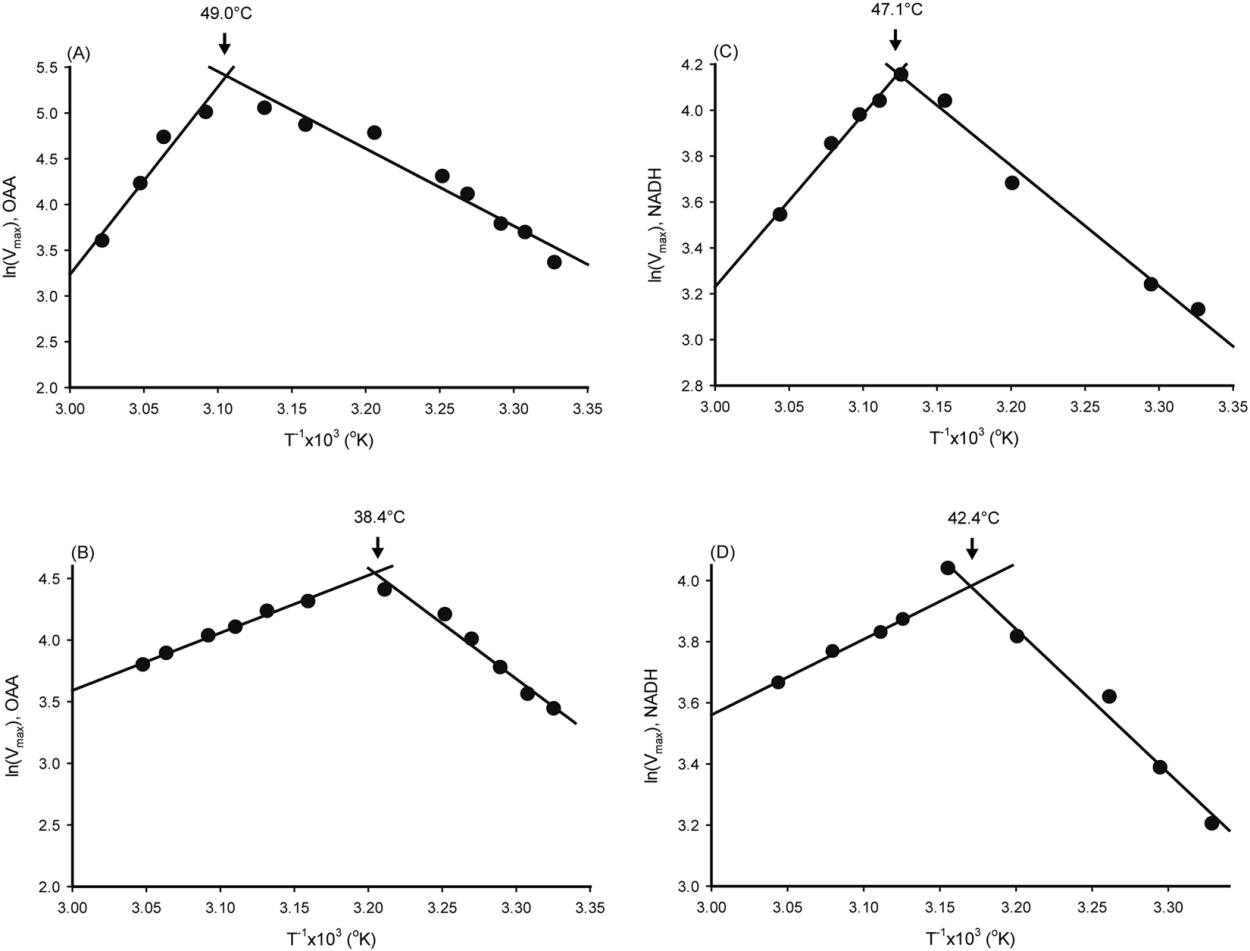
Arrhenius plot of *V*_max_ on temperature for estimating the optimum temperature of malate dehydrogenase from *Platygyra verweyi* with different substrate specificities, OAA (**A,B**) and NADH (**C,D**), at pH 7.0. (**A**) and (**C**) are the enzyme of corals from NPP-OL, (**B**) and (**D**) is from WLT.

**Figure 4:**
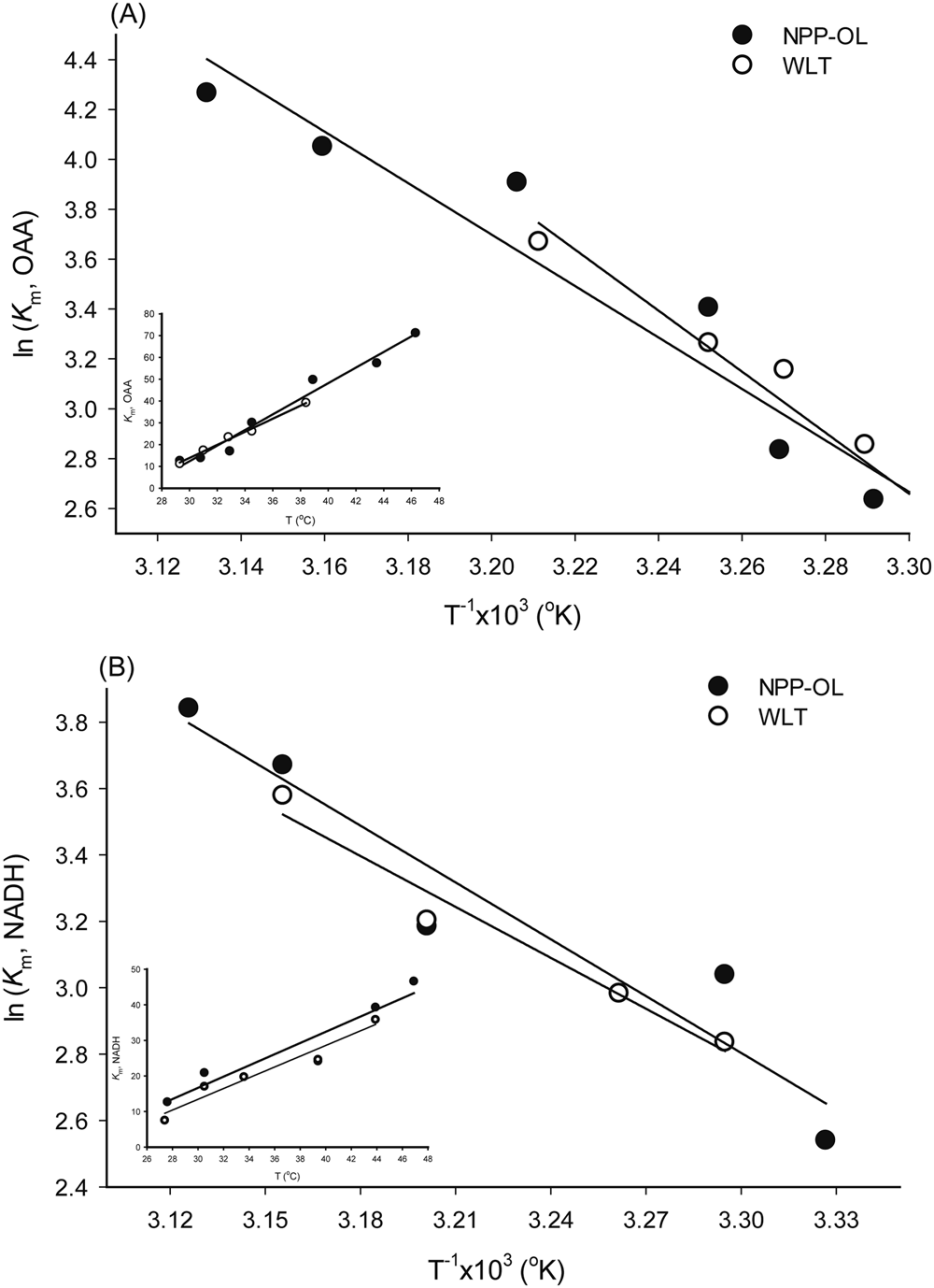
Effect of temperature on the apparent *K*_m_ of pre-inactivated malate dehydrogenase from *Platygyra verweyi* at pH 7.0. Conditions for determining apparent *K*_m_ of pre-inactivated malate dehydrogenase are described as in Fig. 3. Results obtained with various OAA concentrations are shown in (**A**) and NADH are shown in (**B**). Results displayed in degree C are also shown in the inset.

The apparent *K*_m_ and *V*_max_ of MDH varied around 27 °C for the samples from NPP-OL and WLT; this was 7.5 and 12.7 µM for the *K*_m_ of NADH, 9.4 and 5.7 µM for the *K*_m_ of OAA, 24.6 and 22.9 nmol min^−1^ for the *V*_max_ of NADH, and 31.2 and 28.9 nmol min^−1^ for the *V*_max_ of OAA. When the reaction temperature rose, the Arrhenius plot with ln(*V*_max_) on T^−1^ in °K showed a linear increase and then decrease at some higher temperatures, as shown in Fig. 3. The data’s fitness to linear regression model and regression parameters are described in Table S1. According to the intersection of the Arrhenius plot in Fig. 3, samples from NPP-OL performed at a 4.7~10.6 °C higher optimum temperature than those from WLT, depending on substrates. However, the activation energy for MDH catalysis (Ea_cat_), calculated from the data in Fig. 3, were as shown in Table 1 for the Ea_cat_ of OAA and NADH, respectively, and did not display significant differences between the two sampling locations (P > 0.05). When plotting ln(*K*_m_) for temperature in T^−1^ (°K) (Fig. S6), *K*_m_ initially displayed a linear increase, but, different from the performance of *V*_max_ in Fig. 3, came to a plateau after the optimum temperature. Therefore, the sensitivity of *K*_m_ to rising temperatures was examined with the data sets obtained from pre-optimum temperatures. The *K*_m_ dataset’s fitness to linear regression model and regression parameters are described in Table S2. In Fig. 4, the slopes of the MDH *K*_m_ values did not differ significantly between sites (P > 0.05), regardless of whether they were plotted with ln(*K*_m_) on T^−1^ (°K) or *K*_m_ on T (°C) (in inset), implying that samples from NPP-OL and WLT had comparable temperature sensitivities.

**Table 1:** Optimum temperature (opt. T.) and activation energy for catalysis (Ea_cat_) and thermal inactivation (Ea_inact_) of malate dehydrogenase from *P. verweyi* from at the nuclear power plant outlet (NPP-OL) and Wanlitong (WLT).

Sampling Location	Opt. T.		Ea_cat_		Ea_inact_	
	OAA	NADH	OAA	NADH	OAA	NADH
	°C		kJ mol^−1^		kJ mol^−1^	
NPP-OL	49.0	47.1	70	44	115	172
WLT	38.4	42.4	84	39	63	94

**Figure 5:**
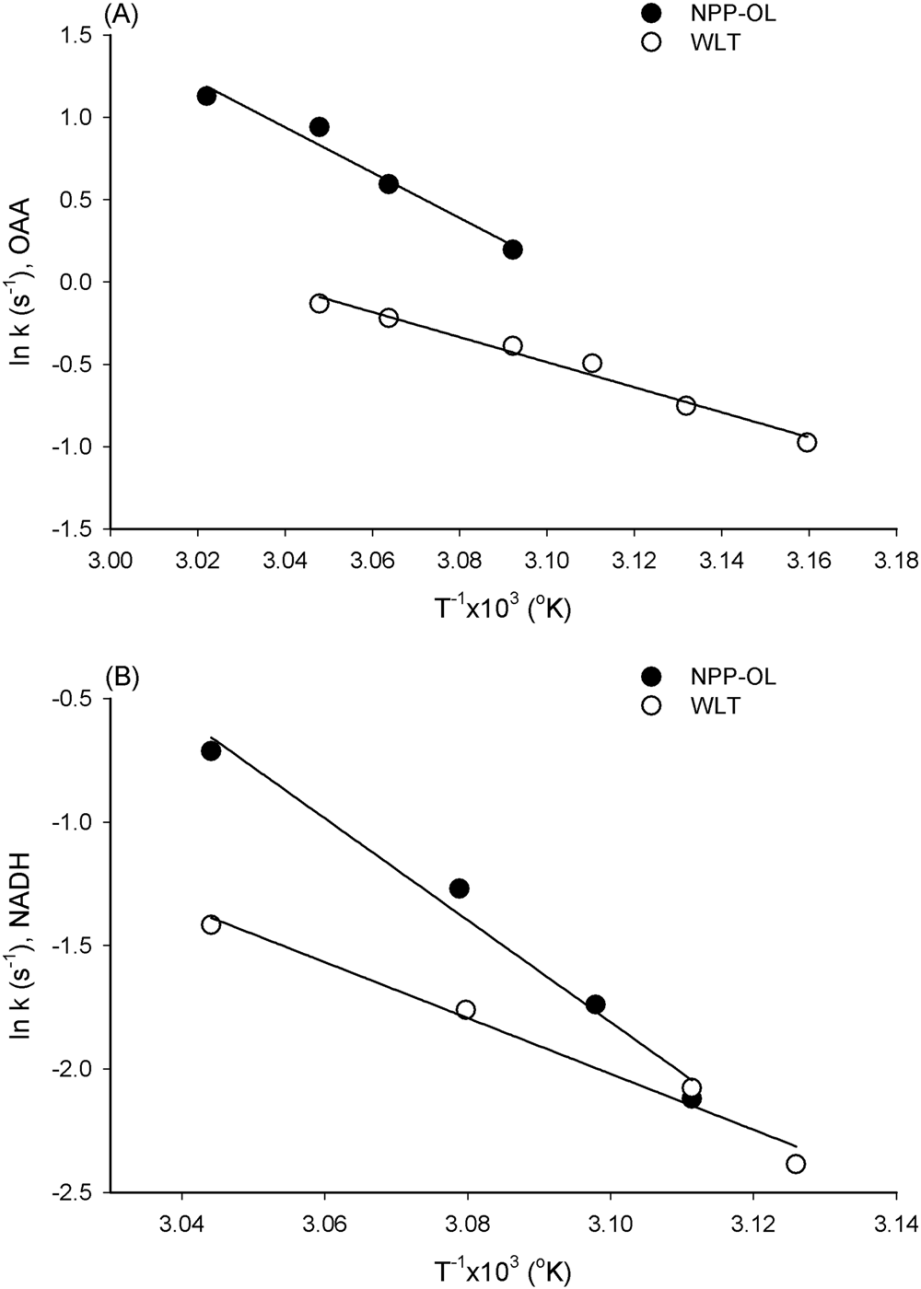
Arrhenius plot for thermal inactivation of malate dehydrogenase from *Platygyra verweyi* at pH 7.0. Conditions for determining apparent *V*_max_ of malate dehydrogenase are described as in Fig. 3. Inactivation rate constant *k* was determined by subtracting the *V*_max_ at each incubation temperature post enzyme inactivation from the *V*_max_ at optimum temperature and converting it to per second.

**Figure 6:**
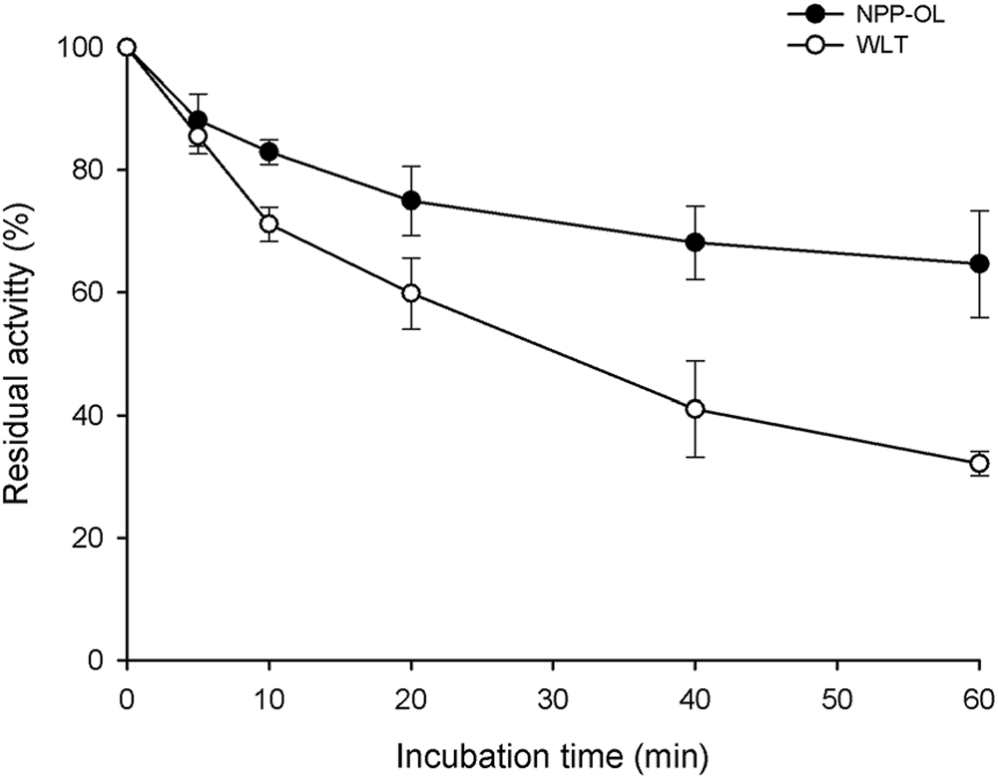
Thermal stability of malate dehydrogenase in *Platygyra verweyi* at pH 7.0. Malate dehydrogenase (ca. 20 nmol min^−1^) obtained from Sephacryl S-200 eluate, as described in Fig. S3, was incubated in a 50 °C water bath for 1 h, and residual enzyme activities were determined periodically.

The activation energy for thermal inactivation (Ea_inact_) and the enzyme stability at 50 °C were investigated to further compare the differences in thermal sensitivity of MDH between NPP-OL and WLT. Ea_inact_ of MDH was determined with the Arrhenius plot of ln(*k*) on T^−1^ (°K), as shown in Fig. 5, in which all four data sets fit the linear regression model well (see regression parameters and coefficients in Table S3). MDH displayed significantly different slopes between NPP-OL and WLT for both substrates (Fig. 5, P < 0.05). Calculated Ea_inact_ indicated that MDH from NPP-OL samples required almost two times higher activation energy to inactivate the enzyme than MDH from WLT, as shown in Table 1. When MDH was incubated at 50 °C, the enzyme activity of the samples from WLT decreased with time faster than that of samples from NPP-OL, as shown in Fig. 6. Moreover, residual activity for the MDH from NPP-OL samples was 64 ± 9% (mean ± S.D., n = 3), which was nearly two times higher than that from WLT (32 ± 2%).

## Discussion

This study clearly demonstrates that coral hosts have the potential to acclimatize and/or adapt physiologically and enzymatically to long-term thermal stress. The 3rd nuclear power plant, operating since 1984, has been discharging thermal effluent from the outlet (NPP-OL), resulting in 2.0–3.0 °C warmer summer water temperatures in the adjacent reef compared to other nearby reefs, such as WLT. The RBT of aposymbiotic *Platygyra verweyi* in NPP-OL was also nearly 3.0 °C higher than that in WLT, suggesting that coral hosts in NPP-OL, despite the high percentage of individuals that associate with thermal-tolerant *Durusdinium spp*^20^, might have also physiologically adapted and/or acclimatized after being impacted by the consistent warmer SST for over 30 years. The critical temperature RBT (also named Arrhenius Breaking Temperature, ABT^43^) for the mitochondria of aquatic organisms is shown to be highly correlated with their maximum habitat or acclimation temperature, and the species adapted to warmer temperatures would perform the RBT closer to that temperature^43^. Consistent with the other aquatic organisms, the higher RBT values in aposymbiotic *P. verweyi* from NPP-OL than from WLT might be a response to the variation in local temperature between two conspecific populations.

Results of MDH bioassays provide some insights, although preliminary, into the protein adaptations that underpins performance differences between the thermal physiologies of *Platygyra verweyi* collected from different temperature regimes. Results clearly indicated that MDH of *P. verweyi* from NPP-OL was more thermally stable than that from WLT. The kinetic analyses showed that the MDH isolated from *P. verweyi* in NPP-OL displays a 4.7 or 10.6 °C higher optimum temperature and 1.8 fold higher Ea of thermal inactivation than that from WLT, depending on substrates. In addition, the MDH of *P. verweyi* from NPP-OL showed 2 times higher residual activity than that from WLT after 1 h at 50 °C. When the enzyme’s adaptation to temperature was examined, the ligand affinity as measured by apparent Michaelis–Menten constants (*K*_m_) was usually modified to respond to environmental temperatures^38,40–42,44,51,59,60^. The MDH was also proposed to be functionally and structurally more sensitive to temperature perturbation than another ATP-generating related enzymes in mussels^50^. However, *K*_m_’s responses to temperature were comparable between the purified *P*. *verweyi* MDH in NPP-OL and that in WLT. Consistent with the response of *K*_m_ to temperature, the catalytic Ea of MDH from *P*. *verweyi* in NPP-OL was not significantly different from that of WLT samples. When the *V*_max_ of MDH from *P*. *verweyi* in NPP-OL was calculated from the equations derived from Fig. 3 with the optimum temperature of that from WLT (42.4 and 38.4 °C for that of NADH and OAA, respectively), the results indicated that the *V*_max_ values were very close between two sites (NPP-OL/WLT: 50/53 and 91/97 for NADH and OAA, respectively). A study on Antarctic notothenioid fish showed that A_4_-lactate dehydrogenase (LDH) decreased activation energy and substrate affinity (i.e., increase in *K*_m_) for adapting to cold temperature^51^, this suggests that, in order to provide comparable *V*_max_ by balancing between stability and flexibility, adaptation to temperature might be achieved by modifying enzyme structure to increase *K*_m_ and decrease catalytic Ea. Similar to the response of *K*_m_ to temperature, catalytic Ea and *V*_max_ at the same temperature between the MDH from NPP-OL and WLT samples suggest that higher thermal resistant performance in the MDH from NPP-OL than WLT samples might not be attributed to temperature adaptation mechanisms described in the literature^6,40–42,44,51,59,60^.

Second, configuration analysis also indicated that the genes coding the MDH of *Platygyra verweyi* may be different from those in published studies in other marine invertebrates^50,59^. Both MDH samples from NPP-OL and WLT consistently showed two distinct MDH fractions with about 25 and 75% of total activity, according to the DEAE profile, suggesting that the different respiration physiology performances might not be attributed to the variations between the two fractions. In addition, only one MDH isoform was obtained in the sample from NPP-OL and WLT, respectively, when the major MDH fraction (peak II) was purified. The purified MDH from both sites were all in the dimer form. In current available coral genome^60,61^, *Stylophora pistillata* contains two MDH isoforms with very similar monomer molecular weights (33.6 and 36.1 kDa) within at least 4 MDH genes^61^. However, the genome data could not manifest if these monomers were constructed into dimer as found in the MDH sample from *P. verweyi*. Although in the literature, MDH adaptation was shown to be achieved with 1 or 2 amino acid mutations. A nearly 1 kDa difference in MDH molecule between that from NPP-OL and WLT might be attributed to two alternative possibilities. One possibility is acclimatization scenario with 2 MDH enzymes with very similar molecular weight, but with different thermal stability in the *P. verweyi* from NPP-OL and WLT. Another possibility is adaptation scenario in which MDH from NPP-OL contains roughly 9 amino acid mutations compared to that from WLT. The proposed hypothesis can be tested through direct protein sequencing or cloning the target gene for further DNA sequencing. At present, we do have analysis carried out using MALDI-TOF mass spectrometry and this information will be using in the future studies to clone MDH gene for further sequencing. Therefore, genetic analysis of the genes encoding the MDH should be conducted before we can conclude whether MDH genes of *P. verweyi* from NPP-OL already show signs of acclimatization and/or adaptation.

Even though changes in the algal symbiont from thermal sensitive to tolerant genotypes (e.g., *Durusdinium* sp.) were highlighted as a potential mechanism of coral acclimatizing to high temperature^15,24^, the species compositions of corals in NPP-OL were dramatically changed with long-term exposure to high temperatures and concurrent associations with tolerant *Durusdinium* spp^20^. *P. verweyi* is one of few coral species in NPP-OL that has not only survived, but prospered^19^. If the more thermally stable MDH isoform found in NPP-OL inhabiting *P. verweyi* was a result of selection from the regular thermal stress in the summer, would it be possible to acclimatize a population in WLT to survive in NPP-OL? Reciprocal transplantations of *P. verweyi* between NPP-OL and WLT^54^ suggest that heating period duration was also important to the *P. verweyi* survival rate when transplanted from WLT to NPP-OL. We conducted two reciprocal transplantations in April of 2014 and 2015 so the transplanted corals could have 3~4 months to acclimatize to the warmer water occurring in the summer. The results of the transplantation indicated that the survival rates of *P. verweyi* transplanted from WLT to NPP-OL depends highly on the duration of the warm period^54^. If the accumulating heat stress is prolonged beyond a threshold—for example, 10.43 degree of heating week (DHW)—*P. verweyi* would barely survive, even if shuffled from the thermally liable symbiont to stress tolerant *D. trenchii*^54^. Conversely, the NPP-OL colonies transplanted to WLT displayed a higher growth rate than conspecific NPP-OL and WLT native populations. Therefore, in order to understand how coral will adapt to changing climate and increased occurrences of high seawater temperatures, it is important to resolve the puzzle involving mechanisms behind aposymbiotic coral host acclimatization and/or adaptation, symbiont population dynamics and its contribution to coral stress response and the duration of heating period. To completely understand if a given coral species can survive the intensity and duration of temperature stress, insights from aposymbiotic host mechanisms will pay way for a better understanding of the nature of stress resistance and contribution to the same from the coral host perspective.

## Supplementary information

Supplementary information

## Data Availability

All additional data from the experiment are provided in “Supplementary Materials”.
