# Supplementary material for: The coral Platygyra verweyi exhibits local adaptation to long-term thermal stress through host-specific physiological and enzymatic response
Source: Sci Rep. 2019 Sep 17;9:13492. doi: 10.1038/s41598-019-49594-z (PMC6748984; doi:10.1038/s41598-019-49594-z)
Supplement: Supplementary file 1 — Supplementary information [file 41598_2019_49594_MOESM1_ESM.docx]

**The coral *Platygyra verweyi* exhibits local adaptation to long-term thermal stress through host-specific physiological and enzymatic response**

Jih-Terng Wang^1*^, Yu-Ting Wang^1^, Shashank Keshavmurthy^2^, Pei-Jei Meng^3,4^, Chaolun Allen Chen^2,5,6*^

^1^Department of Biotechnology, Tajen University, Pingtung 907, Taiwan

^2^Biodiversity Research Center, Academia Sinica, Taipei 115, Taiwan

^3^National Museum of Marine Biology and Aquarium, Pingtung 944, Taiwan

^4^Institute of Marine Biodiversity and Evolution, National Dong Hwa University

Pingtung 944, Taiwan

^5^Institute of Oceanography, National Taiwan University, Taipei 108, Taiwan

^6^Department of Life Science, Tunghai University, Taichung 404, Taiwan

^*^Corresponding authors: J.T.W. E-mail: [jtw@tajen.edu.tw](mailto:jtw@tajen.edu.tw)

C.A.C. E-mail: [cac@gate.sinica.edu.tw](mailto:cac@gate.sinica.edu.tw)

**Supplementary tables and figures**

**Table S1**. Parameters for the linear regression of ln (Vmax) on reciprocal of absolute temperature (T^-1^×10^3^), as described in **Fig. 3**.

| Coral source | Sub. | Increase in rx. rate | | |  | Decrease in rx. rate | | |
| --- | --- | --- | --- | --- | --- | --- | --- | --- |
|  |  | Slope | Y intercept | r^2^ |  | Slope | Y intercept | r^2^ |
| NPP-OL | OAA | -8438 | 32 | 0.951 |  | 20518 | -58 | 0.949 |
| WLT | OAA | -10055 | 37 | 0.994 |  | 4670 | -10 | 0.991 |
| NPP-OL | NADH | -53 | 21 | 0.989 |  | 75 | -19 | 0.985 |
| WLT | NADH | -47 | 19 | 0.986 |  | 25 | -4 | 0.994 |

**Table S2**. Parameters for the linear regression equations of *K*_m_ on temperature, as described in **Fig. 4**.

| ln (*K*_m_) vs °K^-1^×10^3^ | | | | |
| --- | --- | --- | --- | --- |
| Coral source | Sub. | Slope | Y intercept | r^2^ |
| NPP-OL | OAA | -10.32 | 36.72 | 0.936 |
| WLT | OAA | -12.24 | 43.04 | 0.945 |
| NPP-OL | NADH | -5.70 | 21.63 | 0.914 |
| WLT | NADH | -7.65 | 27.76 | 0.857 |
| *K*_m_ vs °C | | | | |
| Coral source | Sub. | Slope | Y intercept | r^2^ |
| NPP-OL | OAA | 3.58 | -94.99 | 0.974 |
| WLT | OAA | 3.00 | -75.95 | 0.992 |
| NPP-OL | NADH | 1.58 | -30.59 | 0.900 |
| WLT | NADH | 1.51 | -31.93 | 0.945 |

**Table S3**. Parameters for the linear regression of ln *k* (s^-1^) on reciprocal of absolute temperature (T^-1^×10^3^), as described in **Fig. 5**.

| Sampling location | Substrate | Slope | Y intercept | r^2^ |
| --- | --- | --- | --- | --- |
| NPP-OL | OAA | -13791 | 43 | 0.968 |
| WLT | OAA | -7608 | 23 | 0.980 |
| NPP-OL | NADH | -20641 | 62 | 0.982 |
| WLT | NADH | -11313 | 33 | 0.977 |

**
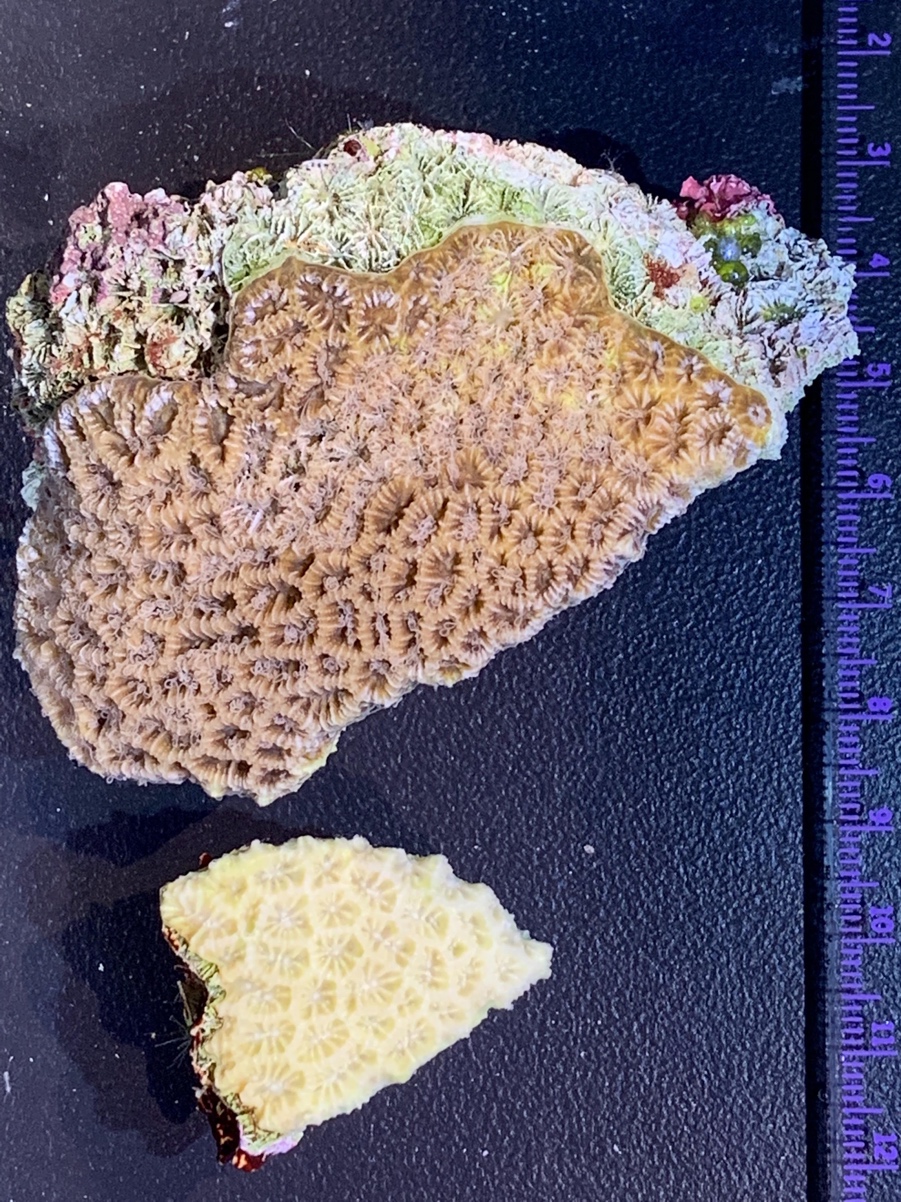
Figure S1.** Photograph of healthy (left) and menthol-bleached (right) *Platygyra verweyi* fragments. Symbiont cells were completely removed from coral samples from both NPP-OL and WLT.


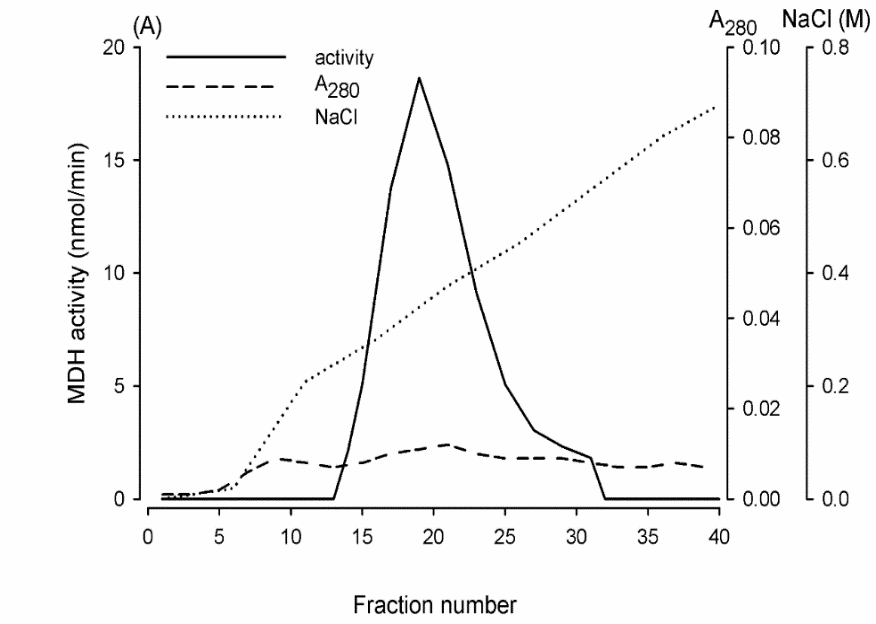

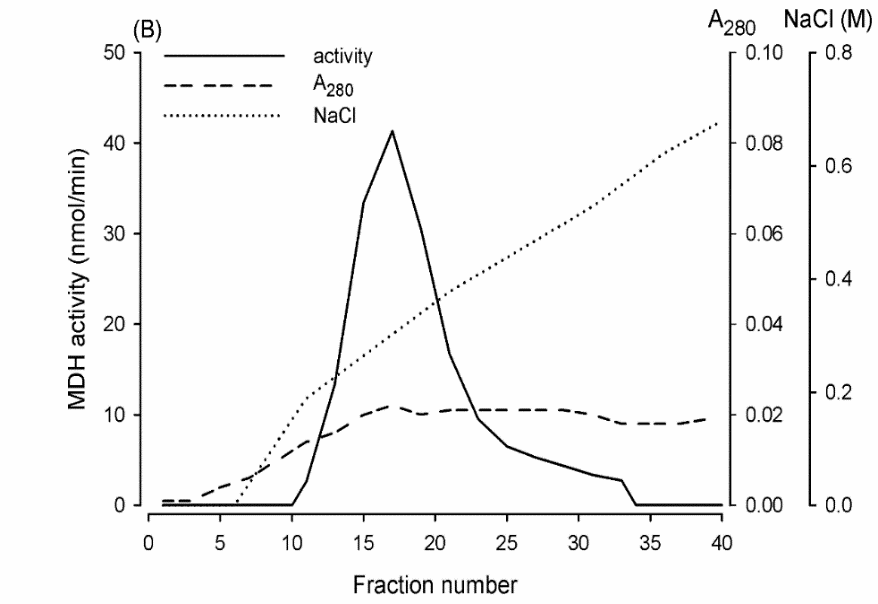


**Figure S2**. Sepharose CL-6B chromatography of major malate dehydrogenase fraction in DEAE eluate, as described in **Fig. 2**. DEAE eluate (adjusted to pH 7.0) was applied to a Sepharose CL-6B column (1×15 cm) equilibrated with 50 mM phosphate buffer (pH 6.6). After being washed with 1 volume of equilibrium buffer, the enzyme was eluted with a NaCl gradient (0.1~0.8 M) at 1 ml min^-1^ under 4°C, and each 2 ml eluate was collected. An enzyme sample of the coral collected from the nuclear power plant outlet (NPP-OL) is shown in (A) and that from Wanlitong (WLT) is in (B).


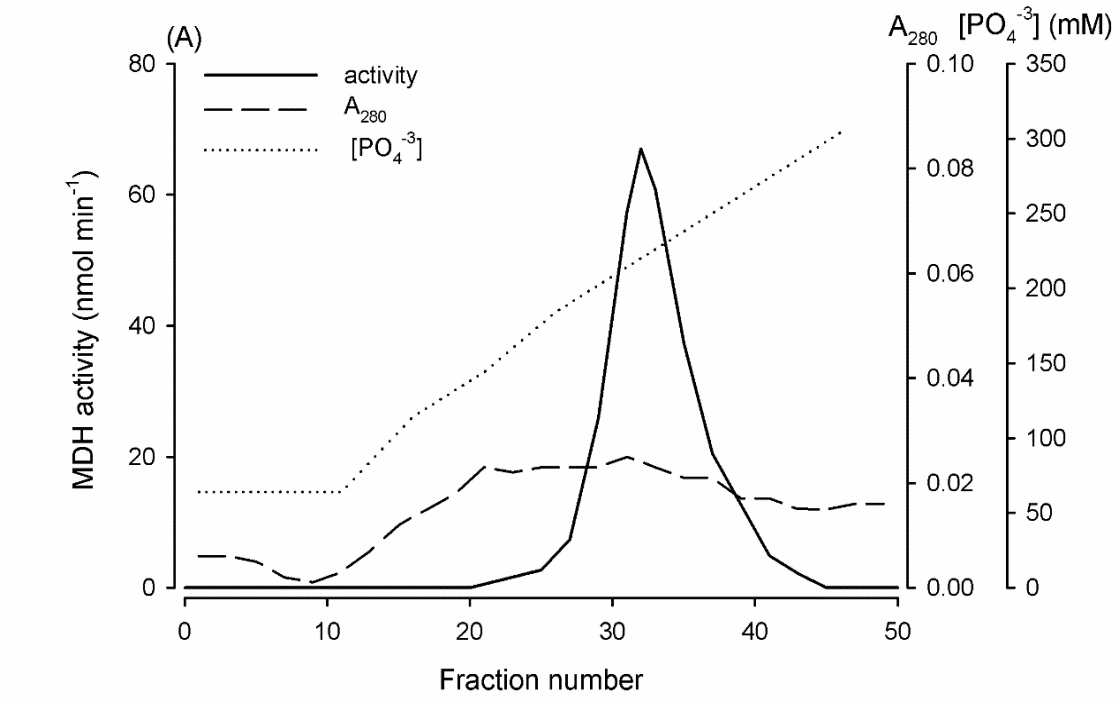

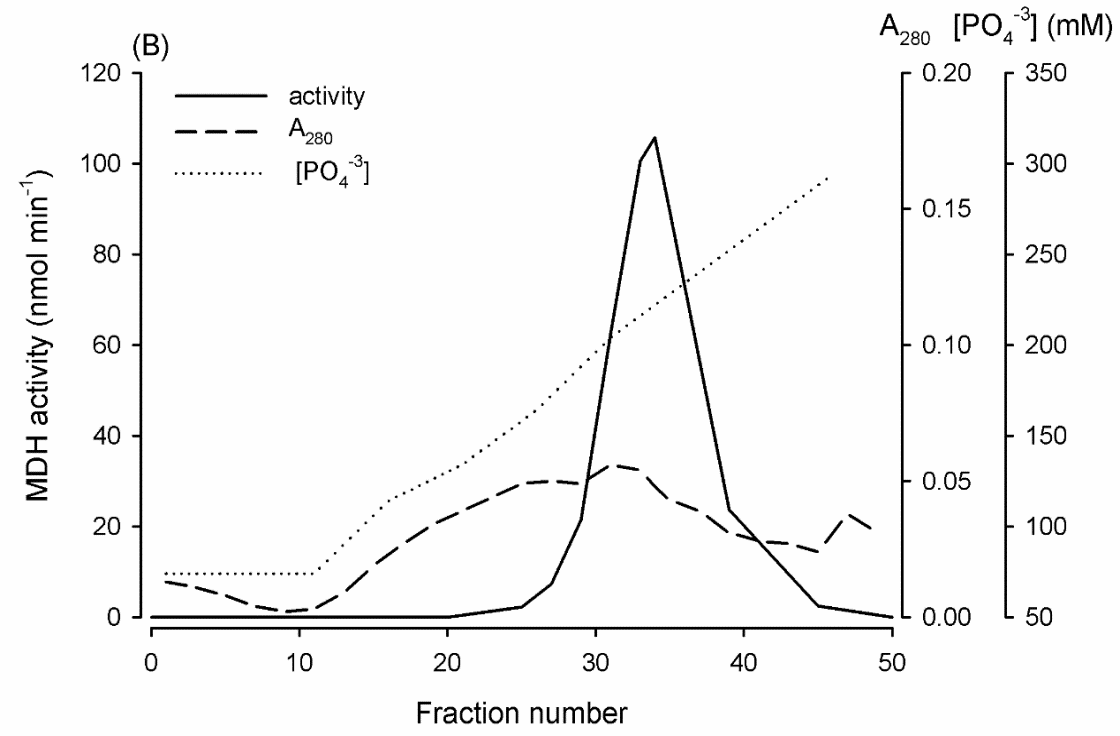


**Figure S3**. Hydroxyapatite chromatography of malate dehydrogenase in CL-6B column eluate, as described in **Fig. S1**. The Sepharose CL-6B eluate obtained from Fig. S1 was directly applied to a hydroxyapatite column (2.6×4 cm) equilibrated with 50 mM phosphate buffer (pH 6.8). After being washed with 1 volume of equilibrium buffer, the enzyme was eluted with a phosphate buffer gradient (50~400 mM) at ml min^-1^ under 4°C, and of 2 ml eluate was collected. an enzyme sample of the coral collected from the nuclear power plant outlet (NPP-OL) is shown in (A) and that from Wanlitong (WLT) is in (B).


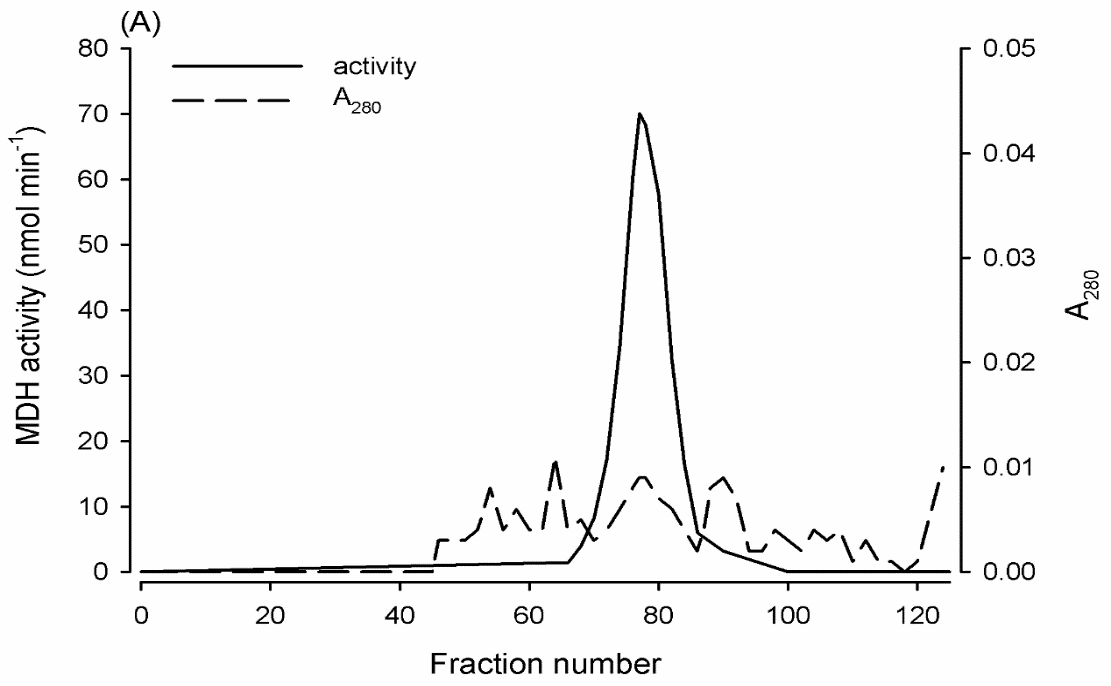

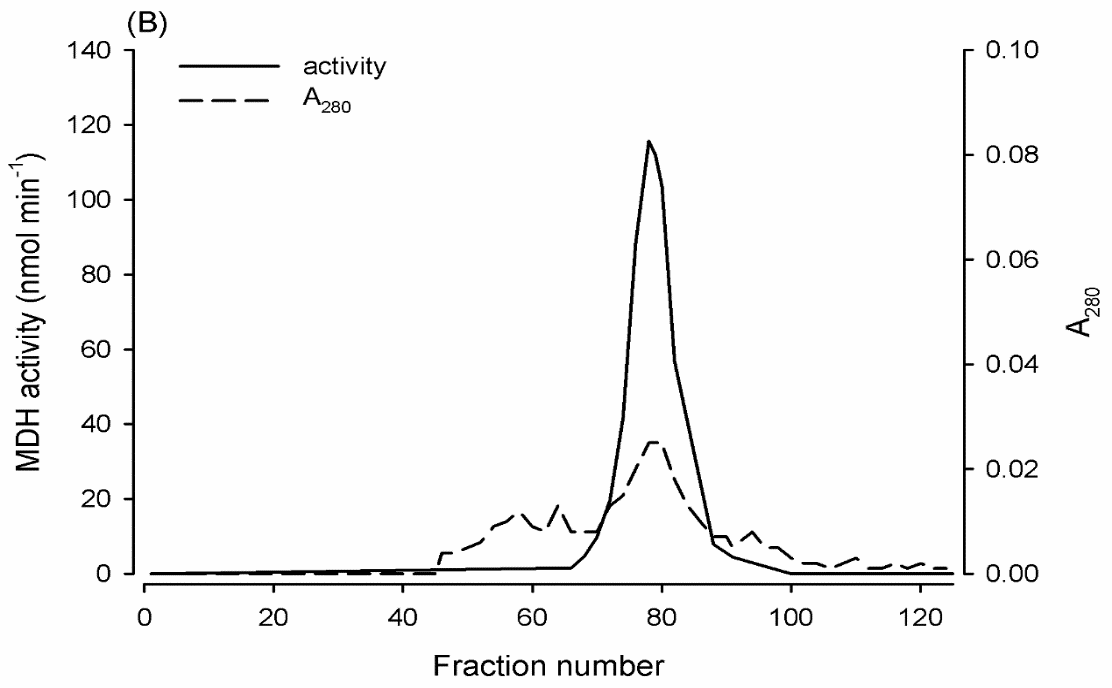


**Figure S4**. Sephacryl S-200 chromatography of malate dehydrogenase in a hydroxyapatite column eluate, as described in **Fig. S2**. About 4 ml of concentrated enzyme sample was subjected to a Sephacryl S-200 column (1.6×91cm) and equilibrated with 10 mM Tris-HCl (pH 8.1) containing 150 mM NaCl at 0.4 ml min^-1^ under 4°C, and each 1.2 ml eluate was collected. An enzyme sample of the coral collected from NPP-OL is shown in (A) and that from WLT is in (B).

**Figure S5**. SDS-PAGE of malate dehydrogenase in Sephacryl-S200 eluate, as described in **Fig. S3**. About 2 µg desalted malate dehydrogenase purified from *Platygyra verweyi* collected from NPP-OL or WLT was separated in 12.5% polyacrylamide gel and visualized with coomassie blue stain. Separate gels were run for samples from NPP-OL and WLT and combined for clarity. The individual gels (for NPP-OL and WLT stained with coomassie blue stain) is included at the end of this file for reference.


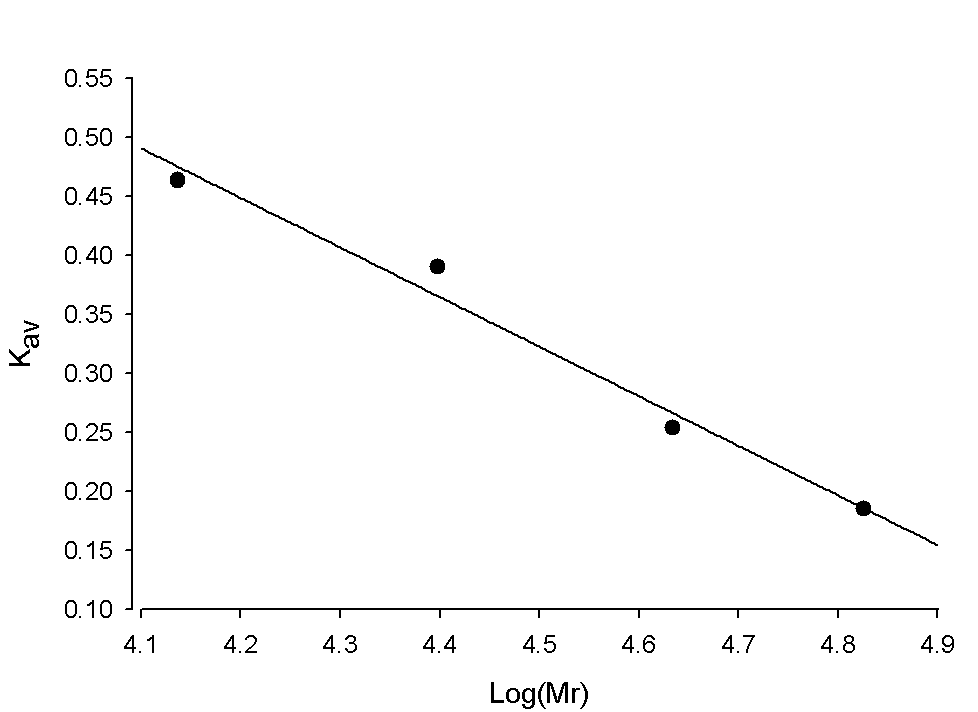


Ribonuclease A

Chymotrypsinogen A

Ovalbumin

Albumin

## **Figure S6**. Standard curve for molecular weight determination of malate dehydrogenase in Sephacryl S-200 eluate, as described in **Fig. S4**. Two ml of protein sample was chromatographed with a Sephacryl S-200 column, as described in **Fig. S4**. The molecular weight calibration curve obtained from standard proteins (ribonuclease A, 13.7 kDa; chymotrypsinogen A, 25 kDa; ovalbumin, 43 kDa; albumin, 66.5 kDa) was Y = -0.4203X+2.2137 (r^2^ = 0.981). The calculated K_av_ was 0.185 (Mw. = 67.0 kDa) for the MDH from NPP-OL and 0.195 (Mw. = 63.5 kDa) for that from WLT.


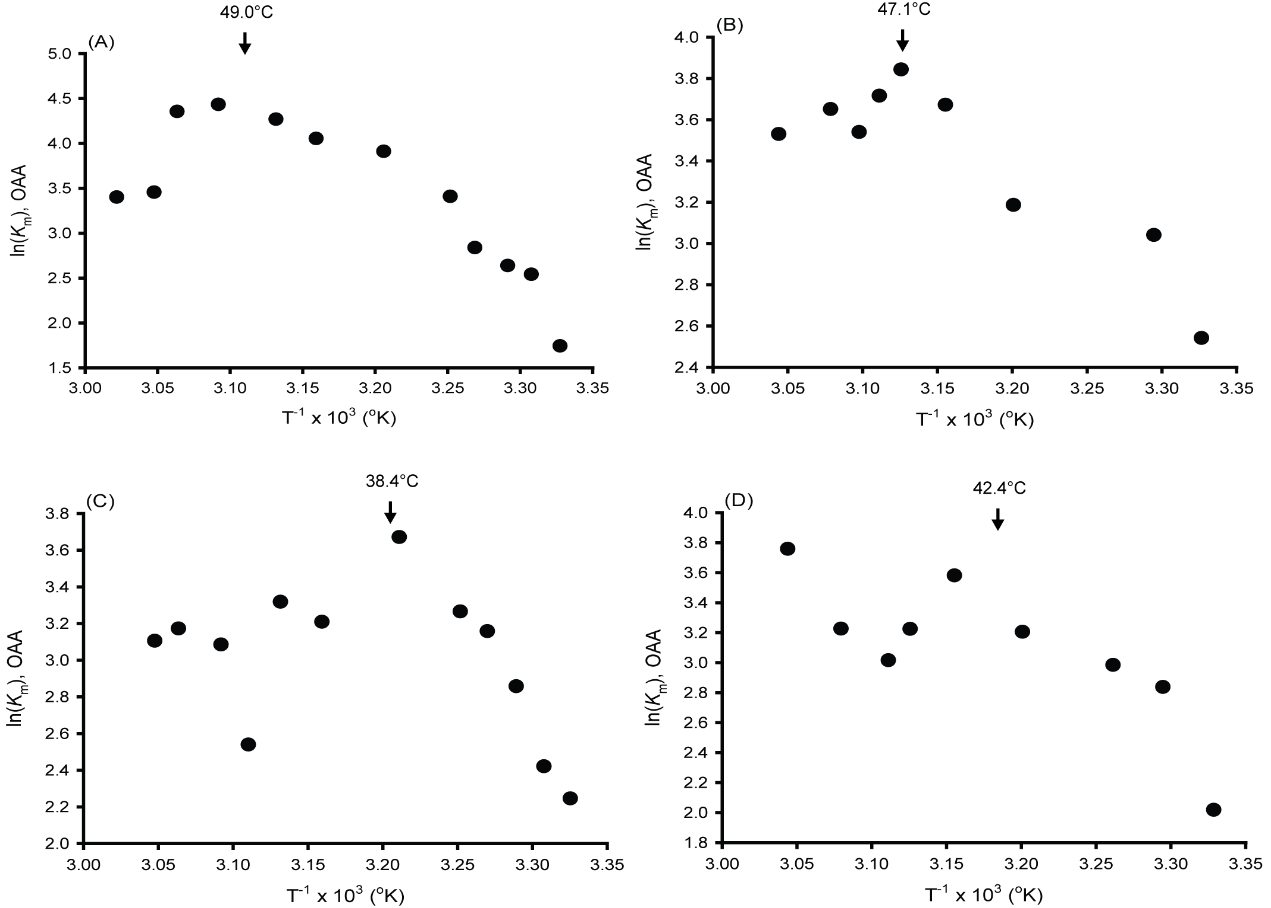


**Figure S7**. Arrhenius plot of *K*_m_ on temperature for evaluating the thermal sensitivity of malate dehydrogenase from *Platygyra verweyi* with different substrate specificities at pH 7.0. The malate dehydrogenase used for kinetic analysis was Sephacryl S-200 eluate, as described in **Fig. S3**. The NADH concentration was fixed at 3mM when determining apparent *K*_m_ with varied oxaloacetate concentration, and at 1 mM of oxaloacetate when NADH concentration was changed. (A) and (C) are the enzymes of coral from NPP-OL, and (B) and (D) are those from WLT.


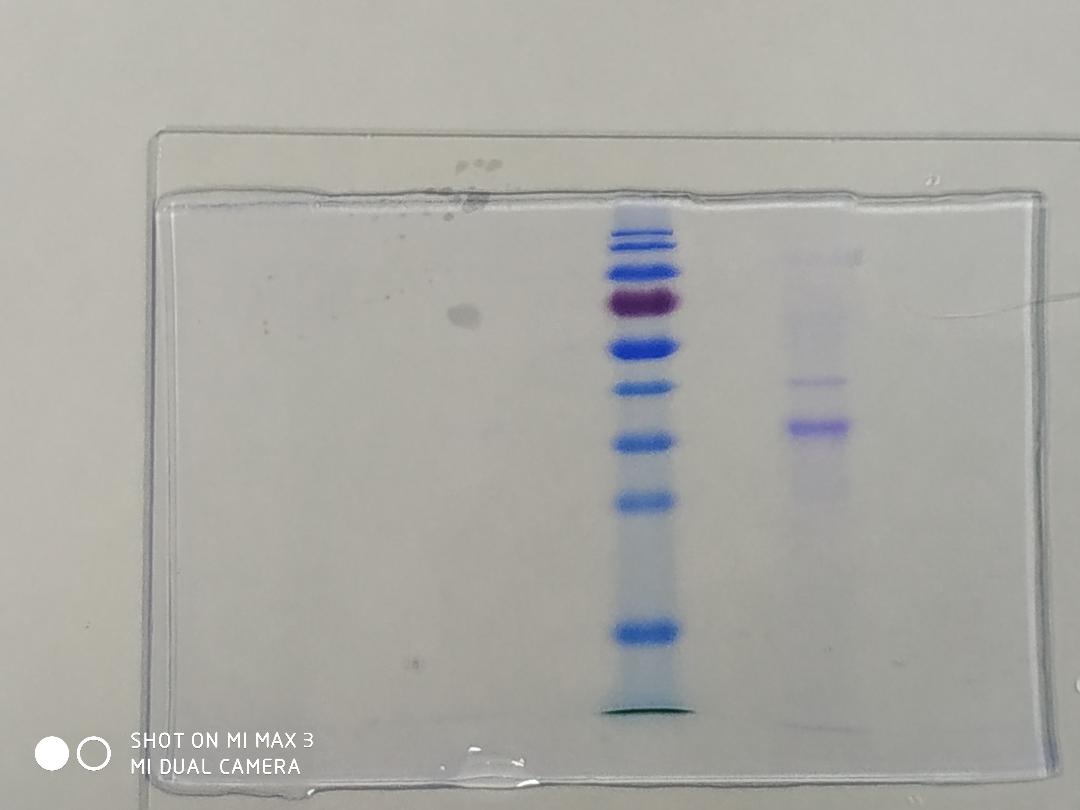


Marker

A

NPP-OL

WLT

Marker


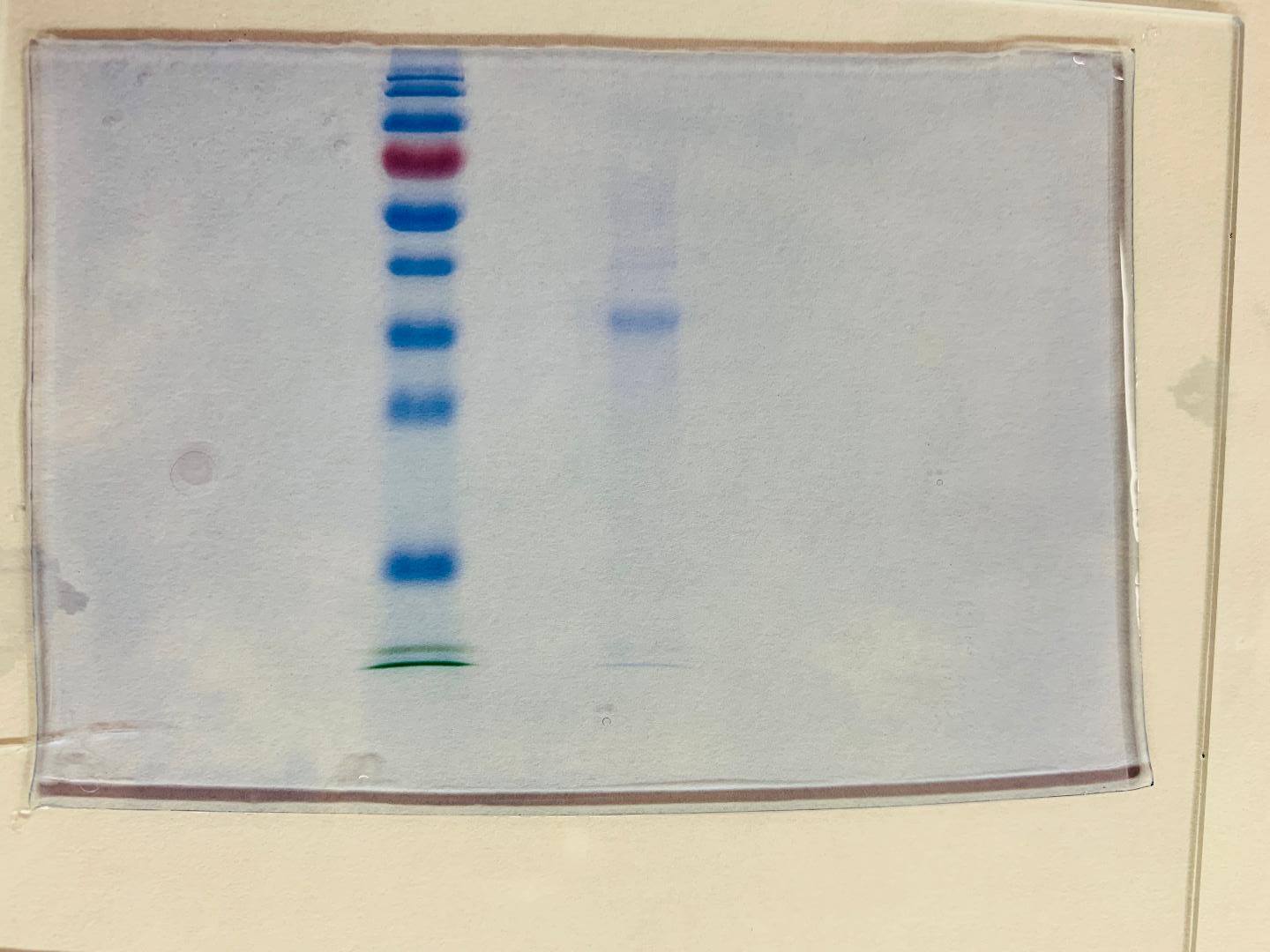


B

SDS-PAGE of malate dehydrogenase in Sephacryl-S200 eluate, as described in **Fig. S3**. About 2 µg desalted malate dehydrogenase purified from *Platygyra verweyi* collected from NPP-OL or WLT was separated in 12.5% polyacrylamide gel and visualized with coomassie blue stain. Separate gels were run for samples from NPP-OL (A) and WLT (B) and combined for clarity for **Figure 5**. The individual gels (for NPP-OL and WLT stained with coomassie blue stain) is included at the end of this file.
